# Local restoration of dystrophin expression with the morpholino oligomer AVI-4658 in Duchenne muscular dystrophy: a single-blind, placebo-controlled, dose-escalation, proof-of-concept study

**DOI:** 10.1016/S1474-4422(09)70211-X

**Published:** 2009-10

**Authors:** Maria Kinali, Virginia Arechavala-Gomeza, Lucy Feng, Sebahattin Cirak, David Hunt, Carl Adkin, Michela Guglieri, Emma Ashton, Stephen Abbs, Petros Nihoyannopoulos, Maria Elena Garralda, Mary Rutherford, Caroline Mcculley, Linda Popplewell, Ian R Graham, George Dickson, Matthew JA Wood, Dominic J Wells, Steve D Wilton, Ryszard Kole, Volker Straub, Kate Bushby, Caroline Sewry, Jennifer E Morgan, Francesco Muntoni

**Affiliations:** a The Dubowitz Neuromuscular Centre, University College London Institute of Child Health London, UK; b Department of Pediatrics, Hammersmith Hospital, London, UK; c Department of Orthopaedics, St Mary's Hospital, London, UK; d Institute of Human Genetics, Newcastle University, Newcastle, UK; e DNA Laboratory Genetics Centre, Guy's Hospital, London, UK; f Department of Cardiology, Hammersmith Hospital Campus, Imperial College London, UK; g Division of Neuroscience and Mental Health, Hammersmith Hospital Campus, Imperial College London, UK; h Robert Steiner MRI Unit, Hammersmith Hospital Campus, Imperial College London, UK; i Academic Unit of Child and Adolescent Psychiatry, Imperial College St Mary's Campus, London, UK; j Hammersmith Hospital Campus, Imperial College London, UK; k Royal Holloway University of London, UK; l Department of Physiology, Anatomy and Genetics, University of Oxford, UK; m Centre for Neuromuscular and Neurological Disorders, University of Western Australia, Perth, WA, Australia; n AVI Biopharma, Corvallis, Oregon, USA; o Centre for Inherited Neuromuscular Disorders, Robert Jones and Agnes Hunt NHS Trust, Oswestry, UK

## Abstract

**Background:**

Mutations that disrupt the open reading frame and prevent full translation of *DMD*, the gene that encodes dystrophin, underlie the fatal X-linked disease Duchenne muscular dystrophy. Oligonucleotides targeted to splicing elements (splice switching oligonucleotides) in *DMD* pre-mRNA can lead to exon skipping, restoration of the open reading frame, and the production of functional dystrophin in vitro and in vivo, which could benefit patients with this disorder.

**Methods:**

We did a single-blind, placebo-controlled, dose-escalation study in patients with DMD recruited nationally, to assess the safety and biochemical efficacy of an intramuscular morpholino splice-switching oligonucleotide (AVI-4658) that skips exon 51 in dystrophin mRNA. Seven patients with Duchenne muscular dystrophy with deletions in the open reading frame of *DMD* that are responsive to exon 51 skipping were selected on the basis of the preservation of their extensor digitorum brevis (EDB) muscle seen on MRI and the response of cultured fibroblasts from a skin biopsy to AVI-4658. AVI-4658 was injected into the EDB muscle; the contralateral muscle received saline. Muscles were biopsied between 3 and 4 weeks after injection. The primary endpoint was the safety of AVI-4658 and the secondary endpoint was its biochemical efficacy. This trial is registered, number NCT00159250.

**Findings:**

Two patients received 0·09 mg AVI-4658 in 900 μL (0·9%) saline and five patients received 0·9 mg AVI-4658 in 900 μL saline. No adverse events related to AVI-4658 administration were reported. Intramuscular injection of the higher-dose of AVI-4658 resulted in increased dystrophin expression in all treated EDB muscles, although the results of the immunostaining of EDB-treated muscle for dystrophin were not uniform. In the areas of the immunostained sections that were adjacent to the needle track through which AVI-4658 was given, 44–79% of myofibres had increased expression of dystrophin. In randomly chosen sections of treated EDB muscles, the mean intensity of dystrophin staining ranged from 22% to 32% of the mean intensity of dystrophin in healthy control muscles (mean 26·4%), and the mean intensity was 17% (range 11–21%) greater than the intensity in the contralateral saline-treated muscle (one-sample paired *t* test p=0·002). In the dystrophin-positive fibres, the intensity of dystrophin staining was up to 42% of that in healthy muscle. We showed expression of dystrophin at the expected molecular weight in the AVI-4658-treated muscle by immunoblot.

**Interpretation:**

Intramuscular AVI-4658 was safe and induced the expression of dystrophin locally within treated muscles. This proof-of-concept study has led to an ongoing systemic clinical trial of AVI-4658 in patients with DMD.

**Funding:**

UK Department of Health.

## Introduction

Duchenne muscular dystrophy (DMD) affects 1 in 3500 newborn boys, causing eventually progressive muscle weakness, cardiomyopathy, and respiratory failure. Patients are diagnosed when they are toddlers, become wheelchair-dependent in their early teens, and die in their 20s. With improvements in standards of care, including non-invasive ventilation and glucocorticoid and cardioprotective treatment, many individuals with DMD survive beyond their mid-20s^1^, ^2^ despite having severe and disabling weaknesses.

DMD is caused by the absence of the protein dystrophin. Dystrophin associates with other sarcolemmal proteins of the dystrophin glycoprotein complex and connects the cytoskeleton to the extracellular matrix. The absence of dystrophin reduces the stability of the sarcolemma and increases intracellular calcium influx, which is followed by degeneration of the muscle fibres. Dystrophin is encoded by *DMD*. Deletions (in about 65% of patients), duplications (in about 10% of patients), point mutations (in about 10% of patients), or other smaller rearrangements can disrupt the open reading frame of *DMD*, leading to premature termination of its translation,^3^, ^4^ whereas deletions or duplications that maintain the open reading frame can lead to truncated but functional dystrophin, which underlies the milder disorder Becker muscular dystrophy (BMD).^5^ The spectrum of severity for BMD varies, ranging from difficulties in walking in the late teens to preserved walking ability into late adulthood and a normal lifespan.^6^

**Figure 1:**
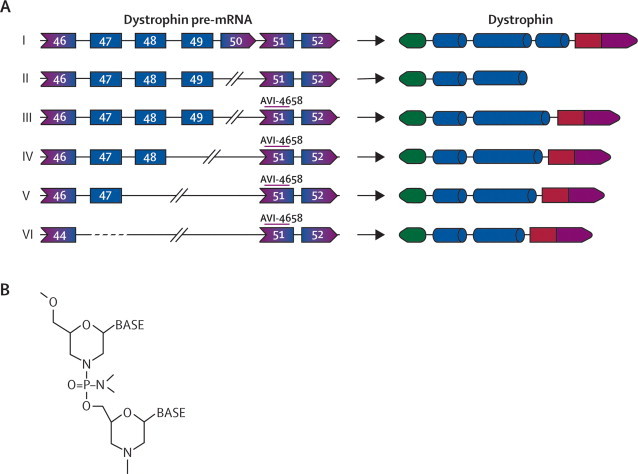
Deletions and predicted results of exon skipping in the patients who were studied (A) Pre-mRNA transcripts and dystrophin protein products from full length *DMD*, in patients with Duchenne muscular dystrophy, and predicted protein sequences after exon skipping. (I) The normal dystrophin gene produces the full length dystrophin product. (II) Patients 1 and 2 had a deletion in exon 50 that disrupts the open reading frame, leading to a truncated and unstable dystrophin. (III) Skipping of exon 51 restores the reading frame, producing a truncated but functional dystrophin that lacks exons 50 and 51. (IV) Patient 7 is missing exons 49 and 50. (V) Patients 3 and 4 are missing exons 48–50. (VI) Patients 5 and 6 are missing exons 45–50. All the truncated dystrophins produced after skipping of exon 51 are missing the hinge 3 region and some of the rod domain but have been associated with the milder BMD phenotype.^9^, ^10^ (B) Structure of the phosphorodiamidate morpholino modification of the antisense oligomer.

Up to 50% of patients with DMD have sporadic dystrophin-positive revertant fibres.^7^ This dystrophin expression arises from alternative processing of *DMD* pre-mRNA that skips some exons, leading to restoration of the open reading frame.^8^ Revertant dystrophin is correctly localised to the sarcolemma and mediates the assembly of other proteins of the dystrophin glycoprotein complex, suggesting that it is physiologically functional. The occurrence of revertant fibres and the mild symptoms of some individuals with BMD with in-frame deletions suggest that it might be feasible to modify the splicing of the *DMD* transcript and, by skipping the mutated exons, produce functional dystrophin (figure 1).

*S*ome exon deletions are more common than others.^11^ Deletions of exons 50, 52, 52–63, 45–50, 47–50, and 49–50 cumulatively account for 13% of all the deletions in *DMD*.^12^ Skipping of exon 51 in patients with these deletions should restore the open reading frame of *DMD* and lead to the expression of functional dystrophin.

Antisense oligonucleotides have been used for experimental gene silencing and recently as splice-switching oligonucleotides to modify splicing and induce exon skipping,^13^ particularly in myoblasts from patients with DMD in vitro,^14^, ^15^ and in mouse and dog models of DMD.^16^, ^17^, ^18^ One patient with DMD who had a deletion of exon 20 received an intravenous infusion of a splice-switching oligonucleotide with a phosphorothioate backbone, which induced skipping of exon 19 and restored the *DMD* open reading frame in lymphocytes but had no effect in skeletal muscle.^19^ A recent phase 1 clinical study reported encouraging results in four boys with DMD who received a single intramuscular injection of a 2*L*'O-methyl-ribooligonucleoside-phoshophorothioate splice-switching oligonucleotide that was targeted to skip exon 51. This treatment led to appreciable expression of dystrophin and was well tolerated.^20^

Other chemically modified oligonucleotides have been used in preclinical models and clinical trials. Phosphorodiamidate morpholino oligomers (PMOs; figure 1) are non-toxic, and in the *mdx* mouse model of DMD they were the most effective oligomer chemistry for inducing exon skipping and restoring long-lasting (weeks) dystrophin expression after intravenous or intramuscular injection.^21^, ^22^, ^23^, ^24^ PMOs, unlike other antisense oligonucleotides, are uncharged, not metabolised, and in preclinical or clinical studies were not associated with activation of the immune system, anaphylaxis, hypotension, or anti-arrhythmias.^25^ On the basis of these data, we have studied the safety and biochemical efficacy of AVI-4658, a PMO designed to target exon 51 that is delivered by intramuscular injection. Here, we report the results of a single-blind, placebo-controlled, dose-escalation safety and efficacy study of PMOs in patients with DMD.

## Methods

### Patients

This single-site, non-randomised, single-blind (investigator) study was done at Imperial College NHS Trust, London, UK, in patients with DMD who were recruited nationally. Participants were boys with a classic clinical diagnosis of DMD^26^ who were aged between 10 and 17 years inclusive when the study drug was given. All participants had a deletion that can be rescued by the skipping of exon 51 (eg, deletion of exons 45–50, 47–50, 48–50, 49–50, 50; 52, or 52–63); had fewer than 5% revertant fibres seen in a muscle biopsy; had the extensor digitorum brevis (EDB) muscle sufficiently preserved (grade 1 to 3: grade 1 is near normal; grade 2 is 30–60% of the muscle is normal; and grade 3 is muscle is almost all abnormal but some normal muscle still present at the periphery), as determined by MRI of the feet;^27^, ^28^ had a forced vital capacity of 25% or more and a normal overnight sleep study before 3 months from the day of injection; were able to comply with all study assessments and return for all study visits; and had adequate psychiatric adjustments, supportive psychosocial circumstances, and full understanding of the study aims, process, and likely outcomes.

Exclusion criteria were: absence of EDB muscles or advanced pathology of EDB muscles (grade 4) on muscle MRI; left ventricular shortening fraction of 25% or less, an ejection fraction of less than 35% seen by echocardiography within 3 months of visit one, or both; respiratory insufficiency defined by the need for invasive or non-invasive ventilation; severe cognitive dysfunction that meant the patient was unable to understand and collaborate with the study protocol; immune deficiency or autoimmune disease; bleeding disorders or chronic anticoagulant treatment within 3 months before study entry; medication with anabolic steroids, creatine protein supplementation, albuterol, or other beta agonists, and intranasal, inhaled, or topical steroids for a disorder other than muscular dystrophy within 1 week before study entry; surgery within 3 months before study entry or planned for anytime during the study; inability to undergo MRI (eg, owing to metal implants); known allergies to products likely to be used in the study (eg, antiseptics or anaesthetics); and participation in another experimental study within 4 weeks of study entry.

Standard-of-care treatment, including glucocorticoids and cardioprotective drugs, was continued in all patients. All study participants were informed before enrolment of the procedures, risks, and possibility of no benefit. All participants provided written assent, and their parents gave written informed consent before enrolment in this study. This trial was designed and done in compliance with UK good clinical practice, International Conference on Harmonisation (ICH) E6, and all applicable regulatory requirements were met (UK Medicines and Healthcare Products Regulatory Agency, UK Gene Therapy Advisory Committee, and local research ethics committees).

Trial activities and adverse events were monitored by a safety monitoring committee. The safety monitoring committee met on the following occasions: before recruitment of the first patient; to authorise the recruitment of the second patient after the first patient was biopsied; and after the second patient was studied but before recruitment of the first patient in the high-dose cohort without use of an intermediate dose (0·27 mg). The safety monitoring committee also met to discuss and authorise a proposed change to the protocol, which enabled us to increase the dose directly to the higher dose, to authorise the recruitment of the last two patients in the high-dose cohort, and to discuss a severe adverse event (bilateral surgical wound infection after the muscle biopsies) in one of the patients in the second cohort.

The protocol was also amended in May, 2008, so that we did not need to recruit the third and last patient into the low-dose group (0·09 mg) or recruit the three patients into the intermediate dose group (0·27 mg), and permitted us to recruit patients in the high-dose group (0·9 mg). Because we had identified considerable comorbidity that precluded recruitment of some of the older patients, we also requested and obtained permission to lower the age at inclusion to 10 years and to be able to recruit ambulant patients.

### Procedures

To confirm that each patient had less than 5% dystrophin-positive revertant fibres, each of the original muscle biopsies used to diagnose the patients was re-evaluated (table 1).^29^ Presence of a deletion suitable for exon 51 skipping and no additional mutations were reconfirmed by sequencing of all the intact *DMD* coding exons and their intron–exon boundaries in all patients. The extensor digitorum brevis (EDB) muscle at the back of the foot was selected as the target muscle. This muscle is well preserved in non-ambulant boys with DMD (Kinali and Muntoni, unpublished), is, for the most part, functionally redundant (an important consideration in a study that is not expected to lead to functional benefit), and can even be absent in some individuals.^30^ MRI confirmed the presence and preservation of the EDB muscles in all patients^31^, ^32^ and that involvement of the EDB muscle was not more than grade 3.^27^, ^28^ Healthy muscle biopsies were obtained from the Dubowitz Neuromuscular Centre biobank.

**Table 1:** Baseline characteristics, exons targeted by PCR primers, and predicted amplicon sizes

	**Age at enrolment (years)**	***DMD* deletion**	**Mobility**	**Steroids**	**Age at first biopsy (years)**	**Dystrophin-positive fibres in original biopsy**	**MRI grading of EDB muscle**	**EDB fibrosis**		**Time between injection and EDB biopsy (weeks)**	**PCR primers to exons**	**Amplicon sizes (bp)**
								Saline injected	Treated			
**Low dose**												
1	16	14 bp deletion in intron 49 that included the exon 50 acceptor splice site	Wheelchair for 11 years	N	8	A few revertant fibres, (∼1–2%); traces on a few fibres	2a	++	++	3	48 and 52	519–286
2	13	Exon 50	Wheelchair for 10 years; rides static bike for 10 min daily	N	7	No revertant fibres; no traces	∞ 2b/3	+++	+++	4	48 and 52	519–286
**High dose**												
3	11	Exons 48–50	Wheelchair for 10 years	Y	7	No revertant fibres; no traces	∞ 2b/3	+++	++	4	46 and 52	570–337
4	15	Exons 48–50	Walks indoors	Y	3	One revertant fibre; traces on many fibres	∞ 2a/2b	+	+	4	46 and 52	570–337
5	11	Exons 45–50	Walks unaided	Y	7	No revertant fibres; traces on a few fibres	1	++	++	4	43 and52	486–253
6	12	Exons 45–50	Walks unaided	Y	3	No revertant fibres; traces on many fibres	2a	++	++	4	43 and 52	486–253
7	10	Exons 49–50	Walks unaided	Y	4	No revertant fibres; traces on many fibres	1	+	+	3	47 and 52	539–306

Numbers are patient number. EDB=extensor digitorum brevis. bp=base pair. Y=yes. N=no. ∞=Asymmetrical EDB involvement on muscle MRI grading. +=Moderate increase of perimysial and endomysial connective tissue; some areas had a severe increase in perimysial and endomysial connective tissue. ++=Most fibres were surrounded by large amounts of connective tissue, but some areas had less and were compact. +++=All fibres surrounded by connective tissue; severe fibrosis throughout sample. EDB=extensor digitorum brevis. bp=base pairs. PCR=polymerase chain reaction

Psychiatric assessments were done to ascertain the expectations and risk of reactive depression for each patient and their family by documenting previous and current psychiatric adjustment and current psychosocial stresses and supports. Parents and children were interviewed separately. The parental questionnaire included the Strengths and Difficulties Questionnaire, the Parental Stress and Support Questionnaire, the General Health Questionnaire, and the Family Assessment Device, which are all validated assessment tools.^33^, ^34^, ^35^, ^36^ Patient interviews with the psychiatrist focused on their understanding of the trial, their general adjustment at home and school, emotional and depressive symptoms, and the Strengths and Difficulties Questionnaire and the Hamilton Anxiety and Depressive Scale.^34^, ^37^

Cultured fibroblasts from a skin biopsy were analysed to verify oligonucleotide-induced splice switching of exon 51 in all patients. Fibroblasts were forced into myogenic differentiation by transduction with an adenovirus expressing the myogenic regulatory factor protein MyoD,^38^ and cultures were transfected with AVI-4658 congener on a 2*L*'O-methyl backbone (300 nM) with Lipofectin (Invitrogen, UK).^39^ RNA was isolated 48 h after transfection and analysed after reverse transcriptase–polymerase chain reaction (RT-PCR) amplification.^39^ 7 days after transfection, cells were harvested for western blot analysis,^39^ and lysates were probed with Dys1 (Vector Laboratories, UK), an anti-dystrophin monoclonal antibody. Dysferlin (Vector Laboratories, UK) was used as a loading control. Baseline safety blood analyses, including tests for anti-dystrophin antibodies^40^ and T-cell subsets (CD4:CD8), were also done.

AVI-4658 is an exon 51-targeted PMO (sequence CTCCAACATCAAGGAAGATGGCATTTCTAG).^39^ AVI-4658 was synthesised and purified by AVI BioPharma (Portland, OR, USA) and was supplied as a low endotoxin and low bioburden powder, which was reconstituted in normal saline in the operating theatre.

This dose escalation intramuscular trial was done in seven patients, who received either of two doses: two patients received 0·09 mg and five patients received 0·9 mg of AVI-4658; both doses were diluted in 900 μL normal saline (0·9%) and were injected in one EDB muscle; the contralateral EDB muscle was injected with 900 μL normal saline. The dose was divided into nine 100 μL injections in the first five patients and four 225 μL injections in the last two patients; this regimen reduced leakage into the skin, which was seen to some extent in patients 1–4. To ensure delivery in the muscle, the drug was injected with a 22 gauge EMG delivery needle (Pajunk, Multistim Sensor, Germany) inside a 1 cm^2^ grid drawn in non-permanent ink on the skin over the site of the EDB muscles. The site and depth of the injections were recorded on videotape. After each injection, the needle was manipulated to confirm its correct placement within the muscle. This took about 1 min. Each infusion was completed in about 30 s. After infusion, the needle was left in place for about 30 s to avoid leakage. The choice of which muscle to inject with the PMO or saline was made in the operating theatre on the day of the injection and the person who made the decision (FM) was masked to whether the patient was right-handed or left-handed. Patients and investigators (except MK, SC, and FM) were masked to which site received the active compound. The procedure was done under general anaesthetic in six patients; one patient opted to have the treatment under local anaesthetic. Both types of anaesthesia were available, and the choice was left to the families.

For all patients, an open biopsy of both EDB muscles was done between 3 and 4 weeks after injection. The rationale for this time frame was taken from previous work in mice and in humans.^17^, ^20^ In the *mdx* mouse, the same level of dystrophin expression was detected at 4 weeks as was detected at 2 weeks after an intramuscular injection of a 2*L*'O-methyl antisense oligonucleotide designed to skip dystrophin exon 23.^17^ Also, in a study of intramuscular injection of a 2*L*'O-methyl antisense oligonucleotide that targeted exon 51, given to patients with DMD, the skipped products and dystrophin were still detected in muscles that were analysed 4 weeks after intramuscular injection.^20^ The area immediately below the needle track was exposed and an open biopsy was taken and rapidly frozen in liquid nitrogen-cooled isopentane, according to standard techniques.^29^ To ensure that all the injection site had been obtained, most of the EDB muscle was removed.

Safety was determined by physical examination and haematological and urinary parameters, which were assessed periodically. The injection sites were monitored for local reaction and reactive pain. Patients were followed-up at timed intervals for 120 days after treatment. Any immune response against the newly synthesised dystrophin was assessed by the production of anti-dystrophin antibodies: serum samples were used to probe western blots loaded with lysates of muscle from the AD17 transgenic mouse, which overexpresses full-length human dystrophin; goat anti-human-IgG was used as the secondary antibody (Bio-Rad, UK).^40^ The presence of T cells and B cells in each biopsy was ascertained by immunohistochemistry with antibodies raised against human CD3, CD4, CD8, or CD20 (Dako, UK).

RNA extraction and RT-PCR analysis were done on ten serial 7 μm sections of the frozen muscle sample.^39^ Direct DNA sequencing of the excised bands was done by University College London Scientific Support Services. For western blotting, proteins from 20 serial 10 μm sections of muscle were isolated directly in 50 μL of loading buffer and analysed as previously described.^39^

For immunohistochemical detection,^29^ unfixed, frozen serial sections (7 μm) were incubated for 1 h with monoclonal antibodies against dystrophin (Dys 2 [exon 77–79]; Vector Laboratories, UK), MANDYS106^41^ (exon 43; a gift from G Morris, Oswestry, and the MDA Monoclonal Antibody Resource), and β-spectrin (Vector Laboratories, UK)^29^ and were then assessed by two investigators (LF and CS) who were masked to the identity of the patient and which side received the active compound. Images were captured with a Leica DMR microscope linked to MetaMorph, version 7.5 (Molecular Devices, CA, USA). Quantitative studies were done as follows: the numbers of dystrophin-positive and dystrophin-negative fibres in the muscle fascicles adjacent to a presumed injection site were counted on the MANDYS106-stained sections of AVI-4658-treated muscles and areas of control muscles chosen at random by two independent investigators (JM and CA). Dystrophin expression was evaluated in 40 muscle fibres selected at random on one representative transverse MANDYS 106-stained region per biopsy. Expression was normalised against the expression of β-spectrin on serial sections and was compared with sections of normal control muscle and the contralateral EDB saline-injected biopsy that were processed in the same way and simultaneously labelled with the same antibodies. Four fields of the immunostained transverse cryosection of each muscle were selected at random (out of focus) and these areas (in focus) were photographed. Ten regions per image, each including an area of membrane and fibre cytoplasm, were selected by moving the cursor across the image and were analysed with MetaMorph. We measured the relative intensity of dystrophin in 100 dystrophin-positive and 100 dystrophin-negative fibres in the same regions of a section of treated muscle from each patient. This trial is registered, number NCT00159250.

### Role of the funding source

The study was funded by the UK Department of Health and sponsored by Imperial College London. Neither had a role in the study design, data collection, data analysis, data interpretation, or writing of the report. AVI Biopharma manufactured and supplied AVI-4658 for the study and provided preclinical testing, packaging, labelling and the investigator brochure for the drug. The company supported the toxicity studies and participated in the design of the protocol, the execution and monitoring of the study, and discussions with the regulatory authorities. All authors have seen and approved the submitted version of the manuscript.

## Results

**Figure 2:**
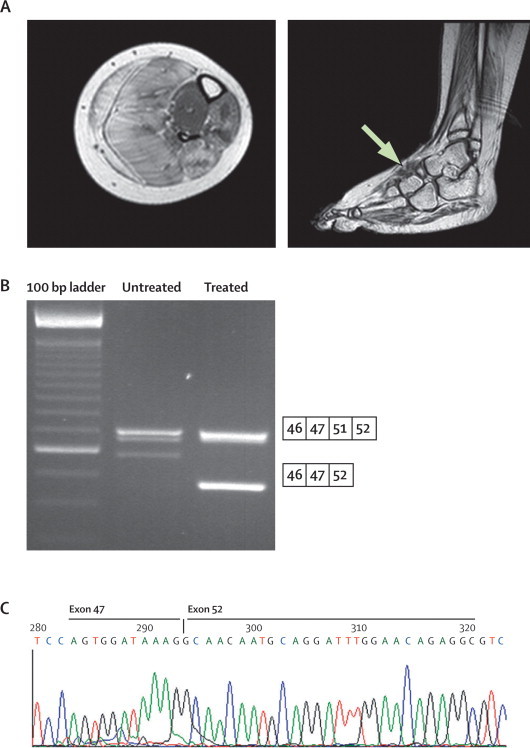
Procedure for prescreening of patients before injection of AVI-4658. Patient 3 is shown as an example; similar results were obtained for all patients. (A) Transverse MRI of the lower leg and coronal MRI of the extensor digitorum brevis muscle (arrow) confirmed the suitability of the muscle. (B) Skin fibroblasts from all patients were forced into myogenic differentiation and treated with an AVI-4658 congener to confirm exon skipping and dystrophin production. RT-PCR analysis shows two bands: the high molecular weight band corresponds to the unskipped transcript (including exons 46, 47, 51, and 52) and the low molecular weight band corresponds to the transcript fragment with size specific skipping of exon 51. (C) Exon 51 skipping was confirmed by sequencing.

MRI confirmed that the EDB muscles in all patients had changes that were less than grade 4 (figure 2). The diagnostic muscle biopsies were re-analysed with Dys1, Dys2, and Dys 3 antibodies to confirm there was no or little dystrophin and less than 5% of the fibres were revertant (table 1). Treatment of MyoD transfected fibroblasts with the 2*L*'O-methyl congener of AVI-4658 showed exon skipping in the RT-PCR products (confirmed by sequencing) and dystrophin expression on western blot in all patients (figure 2, webappendix).

Four patients who had mild cardiac involvement were on treatment with angiotensin-converting enzyme (ACE) inhibitors before recruitment (table 2). Patient psychiatric adjustment and family psychosocial circumstances were deemed to be adequate for all patients included in the study. One patient who was from a family with unrealistic expectations and psychiatric problems was excluded.

**Table 2:** Safety studies and adverse reactions documented during the trial

	**Relative intensity of dystrophin**		**Adverse events**		**Inflammatory infiltrate in muscle**	**Anti-dystrophin antibodies**
	Untreated	Treated	EDB muscle biopsies	AVI-4658 injection		
**Low dose**						
1	9%	6%	Mild bilateral oedema of the forefoot that resolved on day 3	Bilateral mild discomfort, erythema (<25 mm), slight induration, and ecchymosis (>30 mm but ≤50 mm) at the injection sites that resolved on day 3	No difference between the two sides	No difference after injection
2	8%	6%	No local side-effects; decline in cardiac function (FS=22%) but was on ACE inhibitors before the EDB muscle biopsy	Bilateral erythema (50–85 mm) and induration (<25 mm) at the injection sites that resolved on day 3	No difference between the two sides	No difference after injection
**High dose**						
3	11%	22%	No local side-effects; mild biochemical evidence of myoglobinuria, which was self-limiting and resolved after the third micturition after the muscle biopsies	Bilateral mild discomfort, erythema (<25 mm), and slight induration that resolved on day 2	No difference between the two sides	No difference after injection
4	14%	32%	Bilateral ecchymosis that resolved on day 7	Bilateral ecchymosis that resolved on day 3	No difference between the two sides	No difference after injection
5	10%	31%	Mild biochemical evidence of myoglobinuria that was self-limiting and resolved after the third micturition after the muscle biopsies; cellulitis (local pain and redness) in both feet at the sites of the biopsies was treated with a short course of intravenous and then oral antibiotics; refusal to bear weight for 10 days owing to moderate discomfort	Ecchymosis (<20 mm) and slight induration at the AVI-4658 injection site that resolved on day 2	No difference between the two sides	No difference after injection
6	8%	25%	Large ecchymosis (>50 mm) that resolved on day 7	Mild biochemical evidence of myoglobinuria that was self limiting and resolved after three micturitions after the general anaesthetic; mild ecchymosis (<20 mm) on the control foot that resolved after 2 days	No difference between the two sides	No difference after injection
7	4%	22%	No local side-effects or problems	No local side-effects and no problems	No difference between the two sides	No difference after injection

Numbers are patient number. EDB=extensor digitorum brevis. ACEI=angiotensin converting enzyme. FS=shortening fraction.

All safety assessments showed no adverse events that were related to AVI-4568. All patients showed some short-lived, bilateral, localised reactions in both EDB muscles after the injections and muscle biopsies (table 2). In one patient, the echocardiogram at 3 months after the screening visit showed deterioration in fractional shortening, despite ACE inhibitors. This was attributed to the natural history of DMD, and the patient's condition stabilised on beta-blockers. One patient developed bilateral cellulitis in the feet after the muscle biopsies and needed intravenous antibiotics. A short period of refusal to bear weight resolved without consequences.

Light microscopy and immunocytochemistry done masked to treatment showed no differences in inflammatory infiltrates between the treated and control EDB muscles in all patients (table 2). There was no induction of anti-dystrophin antibodies after treatment with the phosphorodiamidate morpholino oligomer, although two patients (patient one and patient six) had low levels of cross reactivity with dystrophin in their pre-treatment and post-treatment serum samples (not shown).

Both patients who had the low dose AVI-4658 showed little expression of dystrophin, despite dystrophin being robustly restored in the cultured fibroblasts, ahead of the injection of the antisense AVI-4658. This suggests that there is a lower threshold effect, and the low dose did not seem to be sufficient to induce exon skipping in patients with Duchenne muscular dystrophy; therefore, we proceeded straight to the high dose.

**Figure 3:**
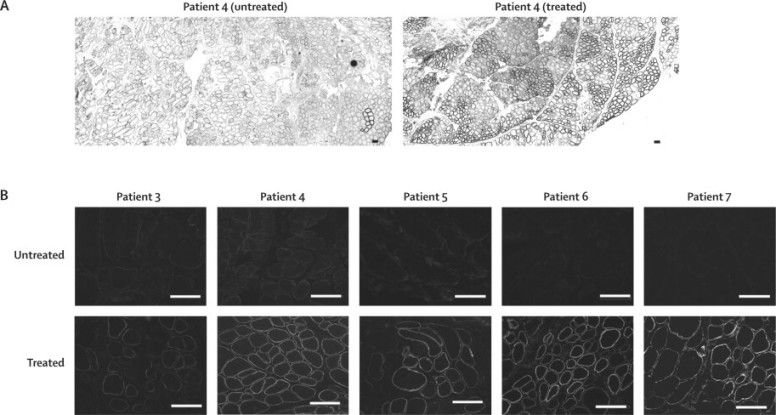
Dystrophin expression in patients treated with high-dose AVI-4658 Transverse sections of treated and contralateral EDB muscles that were immunostained for dystrophin with MANDYS106. (A) Low-power micrograph of a whole section taken with ×10 objective lens shows widespread expression of dystrophin in fibres from the treated muscle in patient 4. (B) Higher magnification (×20 objective lens) of dystrophin immunolabelling in treated and untreated sections in patients 3–7. Scale bars=100 μm.

Biopsies of AVI-4658-injected EDB muscles and the contralateral saline-injected EDB muscles were analysed by assessors who were masked to which muscle was the treated one. This involved quantification of immunostained, dystrophin-positive fibres, the detection of exon 51 skipped RNA (table 1), and immunoblot analysis. After immunostaining of muscle sections with anti-dystrophin antibodies (Dys2 and MANDYS106), all patients treated with high-dose AVI-4658 showed a strong dystrophin signal that prevented masking of which side was the treated one in all patients (figure 3). The results also showed variable low-level immunostaining of the saline-treated muscles in all patients, which underscores the importance of this control (webappendix). Sarcolemmal colocalisation of dystrophin with other proteins of the dystrophin glycoprotein complex (webappendix) suggested that dystrophin interacted with other members of this protein complex and was therefore presumed to be functional. However, in patients one and two, who received low-dose AVI-4658, there was no clear difference in protein expression between the treated and control EDB muscle biopsies (webappendix).

**Figure 4:**
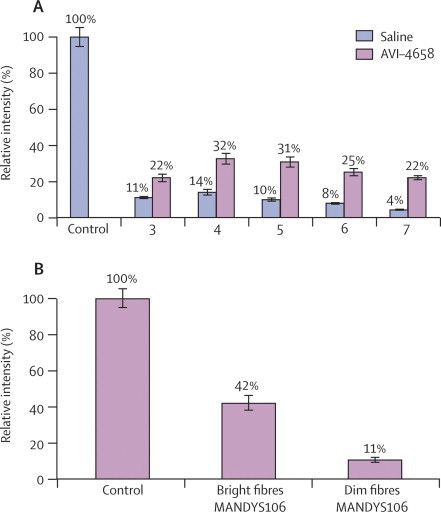
Intensity of dystrophin expression in patients treated with high-dose AVI-4658 relative to control (A) Mean random intensity measurements. (B) Measurement of mean dystrophin intensity in positive fibres: intensity measurements exclusively targeted to 100 dystrophin-positive and 100 dystrophin-negative fibres within the same area in patient 4. Bars are SEM.

The results from the high-dose group were quantified further by image analysis of fluorescent sections stained with the MANDYS106 antibody, which has been used previously for this purpose.^20^ Masked measurements of the fluorescent intensity of dystrophin staining were done in 40 fibres chosen at random per drug-treated and saline-treated muscle sample. In the high-dose group, the mean difference in measurements of the fluorescent intensity of dystrophin expression in all treated muscles was about 17% (range 11–21%) more than that in the contralateral saline-injected muscles (one-sample paired *t* test p=0·002; figure 4). Because the random measurements took into account areas that contained both dystrophin-positive and dystrophin-negative fibres for the intensity measurements, we targeted the dystrophin-positive fibres within the same area. The intensity in these fibres in patient 4 was 42% of that in healthy muscle (figure 4).

Counting of the positive fibres stained with MANDYS106 was done after confirmation of which muscle was the treated muscle and which was the control, owing to low-level staining in several of the patients (Figure 3, Figure 4). If the low-level immunostaining was not factored in, the number of positive fibres in the side that received AVI-4658 reached 100% in several patients. We therefore adjusted the detection threshold so that only the rare revertant fibres were seen in the saline-injected muscle. This threshold was then subtracted from the contralateral AVI-4658 injected muscle. A mean of 419 fibres (range 262–792 fibres) were seen in the four areas counted from the treated muscles in patients from the high-dose group. In the five muscles treated with high-dose AVI-4658 there was a mean of 269·8 (SD 204·5) dystrophin-positive fibres by contrast with 6·6 (8·1) dystrophin-positive fibres in the saline-treated side (one-sample paired *t* test p=0·02). When the proportion of positive fibres in the fascicles that were assumed to relate to the needle track were counted, the number varied between 44% and 79% (mean 59·8% [SD 13·9]; table 3). At least 262 fibres were counted in each sample. There was no correlation between the proportion of dystrophin-positive fibres and the severity of fibrosis (table 1).

**Table 3:** Dystrophin expression in muscle myofibres in patients 3–7, who were treated with high-dose AVI-4658

	**Untreated**		**Treated**	
	Total	Positive	Total	Positive
3	443	21 (5%)	377	182 (49%)
4	662	2 (<1%)	792	623 (79%)
5	475	2 (<1%)	263	116 (44%)
6	554	5 (1%)	404	264 (65%)
7	405	3 (<1%)	262	164 (63%)

**Figure 5:**
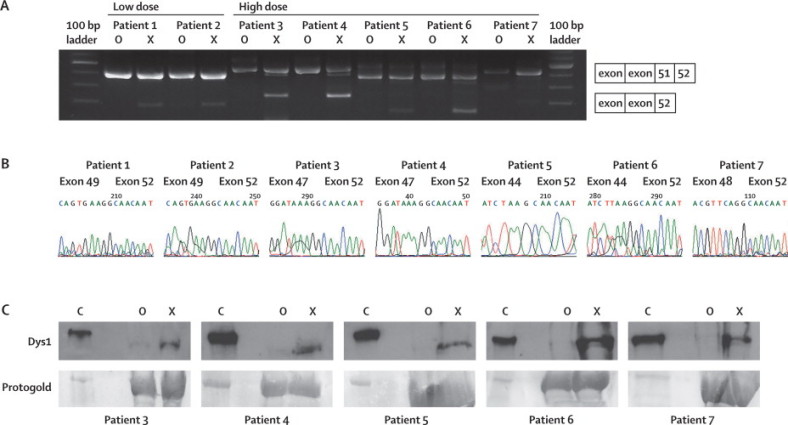
Exon 51 skipping in amplified RNA from treated muscles (A) RT-PCR analysis of RNA extracted from treated (X), untreated (O), and control (C) muscle sections detects shorter transcript fragments in the treated muscles, with sizes that correspond to the specific skipping of exon 51. (B) Exon 51 skipping was confirmed by sequencing. (C) Western blot analysis of homogenates of treated and untreated muscle (20×10 μm sections) and control muscle (2×10 μm sections [to avoid overexposure]) shows dystrophin expression in extracts from the control muscles (C) and treated (X) extensor digitorum brevis but not in the contralateral muscles (O). Loading was monitored with protogold. Low dose=0·09 mg AV-4658. High dose=0·9 mg AV-4658.

The results of the dystrophin immunostaining were corroborated by those from the RT-PCR and western blots, which were done masked to treatment. Exon 51 skipping and distinct bands of dystrophin protein were seen in the drug-treated muscles but not in the saline-treated muscles of patients in the high-dose group (figure 5). Exon 51 skipping was also seen in the two patients in the low-dose group, but this was less abundant and only detected when high-sensitivity conditions were used (additional cycles);^20^ however, these two patients did not have detectable dystrophin on western blot (webappendix). Sequencing of the RT-PCR products confirmed accurate skipping of exon 51 in the treated muscles of all patients (figure 5). Immunoblot analysis showed bands of the expected molecular weight in the AVI-4658-treated muscles.

## Discussion

This single-blind study assessed the local safety and biochemical efficacy of intramuscular injection of AVI-4658 in patients with DMD who have deletions that are responsive to skipping of *DMD* exon 51. We showed, in vivo, that the PMO AVI-4658 induced specific skipping of exon 51 and the production of dystrophin that was correctly localised at the sarcolemma. The treatment was not associated with any systemic or local adverse events or with any immune response against dystrophin.

Specific dose-dependent exon skipping was seen in the treated EDB muscles compared with the contralateral saline-injected muscles. Patients in the low-dose group showed RT-PCR evidence of exon skipping, confirmed by sequencing, but no dystrophin protein was detected.

Strong expression of dystrophin was seen in the treated muscle in all patients in the high-dose cohort after subtraction of the signal intensity for the low-level of antibody labelling in the sarcolemma of the saline-injected contralateral muscle (figure 3). Localisation of dystrophin to the sarcolemma suggests appropriate interaction with other proteins of the dystrophin glycoprotein complex. Western blot analysis detected increased expression of dystrophin in the AVI-4658-treated muscle of all patients who received the high dose, and the immunoblot detected expression of dystrophin of the expected molecular weight in all patients. However, the quantification of dystrophin from a western blot after an intramuscular injection is difficult (eg, ensuring that equivalent amounts of sarcolemmal proteins are loaded in each track, the transfer of large proteins is efficient, and development of the signal in the linear range). In view of the highly localised delivery of AVI-4658, we chose an immunohistochemistry-based method to quantify dystrophin in specific myofibres. This method has several advantages over other techniques, such as western blot and real-time RT-PCR, because it enables the in situ visualisation of the correct localisation of the expressed protein. Additionally, western blot and RT-PCR are not relevant for proteins that are only expressed in a subset of fibres, which is the case here because of the local injection.

The quantitative immunohistochemistry method we used goes a step further than the one used in a recent exon-skipping study,^20^ because fibres are randomly selected to calculate the mean expression in the treated muscle and compared with that in the control muscle (figure 4). We extended our analysis by measuring the intensity of selected dystrophin-positive and dystrophin-negative fibres within the same areas of the treated muscles. We show that the fluorescent intensity of the dystrophin-positive fibres was 42% of that of fibres in healthy, non-dystrophic control muscles (figure 4).

In a recent clinical trial in which exon 51 was targeted, the investigators showed dystrophin expression in the tibialis anterior muscle after one injection of PRO051, a 2*L*'O-methyl antisense oligonucleotide.^20^ Although PRO051 and AVI-4658 target the same region of exon 51, we used a phosphorodiamidate morpholino oligomer chemistry, used a longer splice-skipping oligonuceotide, and treated a small intrinsic and relatively non-functional foot muscle, which enabled us to obtain bilateral muscle biopsies that were not available in the PRO051 study. Because staining with the MANDYS106 antibody, which was used in both studies, results in low-level labelling in some patients with DMD (figure 4), negative controls are crucial for accurate quantification of the protein. This low-level labelling of dystrophin is absent only in patients with DMD in whom the MANDYS106 epitope (coded in exon 43) is deleted (data not shown), implying that a genuine product of *DMD* is detected by this high-affinity antibody. Our controlled study of exon skipping for DMD clearly shows an increase in the expression of dystrophin in drug-treated muscles compared with the saline-injected contralateral muscle. Because a negative saline-injected control was not included in the previous study,^20^ and the background concentration of endogenous dystrophin expression was not taken into account, the relative efficacy of the two compounds for inducing dystrophin-positive fibres cannot be compared directly. If we estimate that we obtained an average dystrophin intensity value of 26·4% (range 22·0–32·0%) in a much larger biopsy (about 0·5×2×2 cm; webappendix) compared with the 27% (range 17–35%) in the previous study, which reported biopsy sizes between 120–726 fibres,^20^ this might suggest that the phosphorodiamidate morpholino oligomer AVI-4658 compares favourably with the 2*L*'O-methyl chemistry.^20^ This seems to be particularly relevant because the method used to measure intensity values in the PRO051 study averaged the intensities of the whole image and did not subtract the low-level background expression. However the methods for quantifying immunocytochemistry in the two studies are not identical, different muscles were studied, and the volume of the injected drug was different; therefore, direct comparison between the two studies cannot be made with precision. This implies that, in terms of the different chemistries used for the antisense oligonucleotides, the efficacies of the results of these two studies cannot be directly compared. Although PMOs that were designed to skip exon 23 were more efficient than were 2*L*'O-methyl oligomers after intramuscular injection in *mdx* mice,^22^, ^24^ any differences in length among the antisense oligonucleotides might contribute to their efficacy.^24^ Nevertheless, both studies reported unequivocal expression of dystrophin at similar concentrations.

Whether this expression of dystrophin resulted in improved muscle function was not studied. However, in-frame deletions of the exons 48–51, 50–51, and 45–51 in *DMD*, which will lead to dystrophin that is similar to that induced in this study, have been described in multi-generation families in which the affected members were asymptomatic.^9^, ^10^ We therefore anticipate that the dystrophin produced by the patients in our study would be functional and speculate that the missing domain is not essential for protein function or structure. The concentration of dystrophin needed to improve or preclude clinical symptoms will probably depend on the quantity and quality (molecular structure) of the protein. We recently reported that dystrophin expression of 27% of the concentration in healthy muscle was sufficient to avoid skeletal muscle symptoms,^42^ but the concentration of truncated dystrophin without exon 51 that is sufficient to provide a clinical benefit is not known. Nevertheless, if the increases in dystrophin concentration that we observed along the needle track were achieved after systemic delivery, then this might lead to a clinically significant response.

Preclinical studies in *mdx* mice have shown that seven weekly doses of PMOs resulted in a high number of dystrophin-positive fibres, varying between 10% and 70% of the muscle fibres in the different muscles analysed.^17^ Translation of dose from intramuscular to systemic studies or studies with the same delivery route but in different species is difficult. However, in a recent study^18^ in which dogs with canine X-linked muscular dystrophy were given an intravenous cocktail of morpholinos designed to skip exons 6 and 8 of dystrophin, expression of the protein was successfully restored and a clinical benefit was noticed without adverse reactions to the high-doses of morpholinos used. This bodes well for systemic studies in humans. On the basis of these observations, we have initiated a dose-ranging study in ambulant patients with DMD to assess the safety and efficacy of repeated doses of systemic intravenous AVI-4658 (ClinicalTrials.gov, number NCT00844597).

## Web Extra Material

Supplementary webappendix
