# Supplementary material for: Local restoration of dystrophin expression with the morpholino oligomer AVI-4658 in Duchenne muscular dystrophy: a single-blind, placebo-controlled, dose-escalation, proof-of-concept study
Source: Lancet Neurol. 2009 Oct;8(10):918–28. doi: 10.1016/S1474-4422(09)70211-X (PMC2755039; doi:10.1016/S1474-4422(09)70211-X)
Supplement: Supplementary webappendix [file mmc1.pdf]

THE LANCET Neurology

Supplementary webappendix

This webappendix formed part of the original submission and has been peer reviewed. We post it as supplied by the authors.

Supplement to: Kinali M, Arechavala-Gomeza V, Feng L, et al. Local restoration of dystrophin expression with the morpholino oligomer AVI-4658 in Duchenne muscular dystrophy: a single-blind, placebo-controlled, dose-escalation, proof-of-concept study. *Lancet Neurol* 2009; published online August 26, 2009. DOI:10.1016/S1474-4422(09)70211-X.

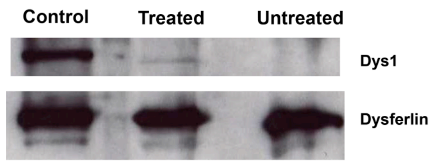

Supplementary Figure 1: **Western blot of cell lysates show dystrophin expression in fibroblasts of patient 3**

Western blot analysis shows dystrophin expression in extracts from the treated, but not in control, non-treated, fibroblasts.

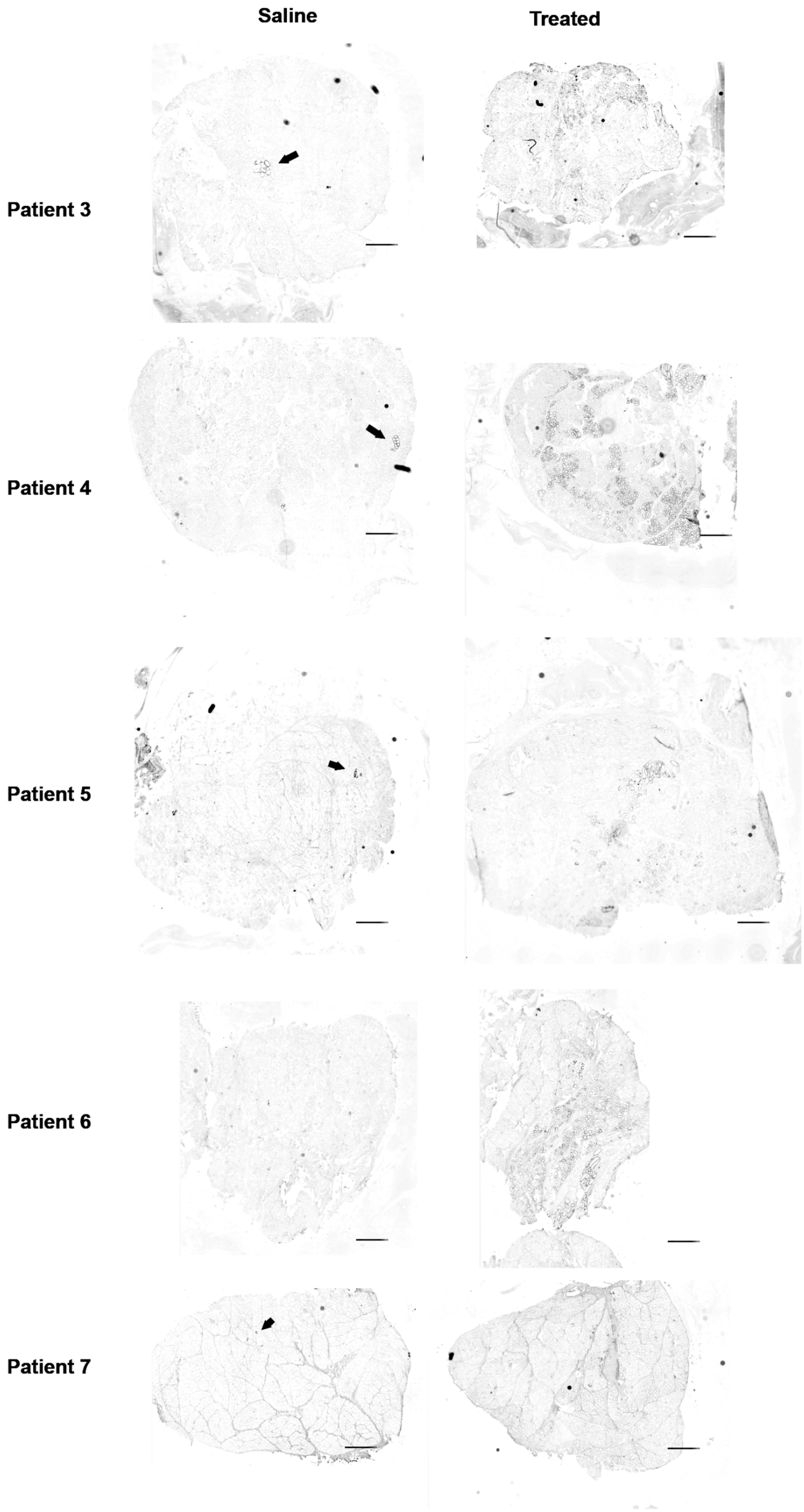
Saline
Treated
Patient 3
Patient 4
Patient 5
Patient 6
Patient 7

***Supplementary Figure 2*: Immunofluorescence analysis of whole sections of the treated and saline injected extensor digitorum brevis muscles show clear sarcolemmal labelling in various muscle fibres.**

Arrows show clusters of revertant fibres in saline-treated samples. Scale bar=1000 µm.

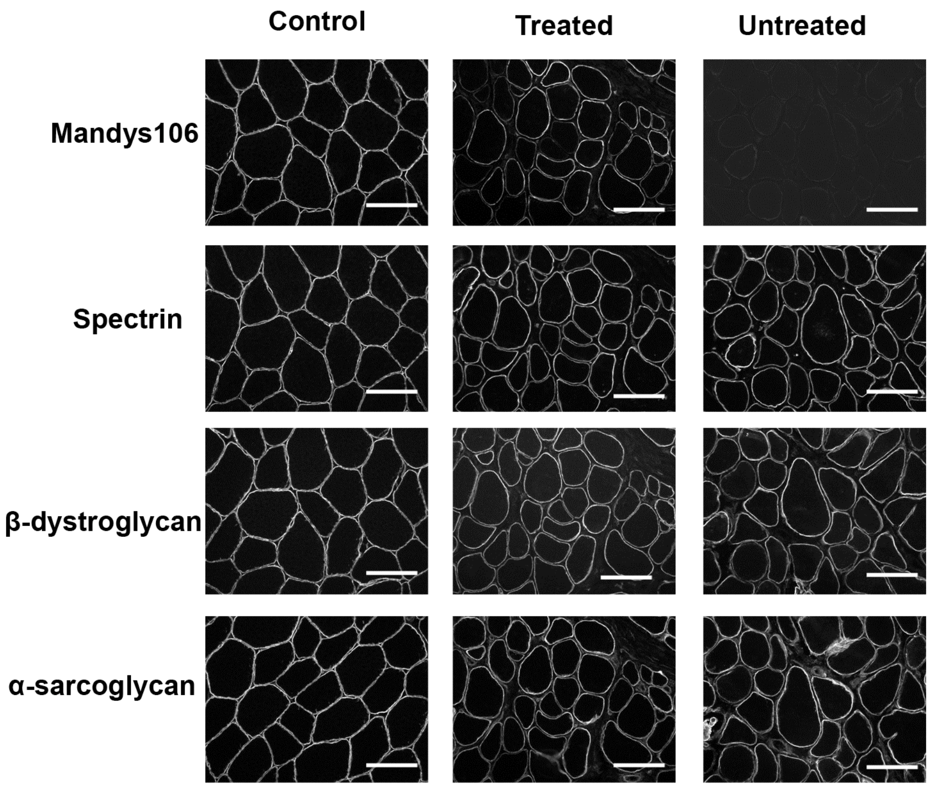

***Supplementary Figure 3*: Serial sections showing sarcolemmal co-localisation of the newly generated dystrophin with dystrophin glycoprotein complex proteins**

Sections from a control, treated, and untreated muscle that show dystrophin (labelled with Mandys106) in the treated muscles co-localised with α-sarcoglycan and β-dystroglycan. Scale bar=100 µm

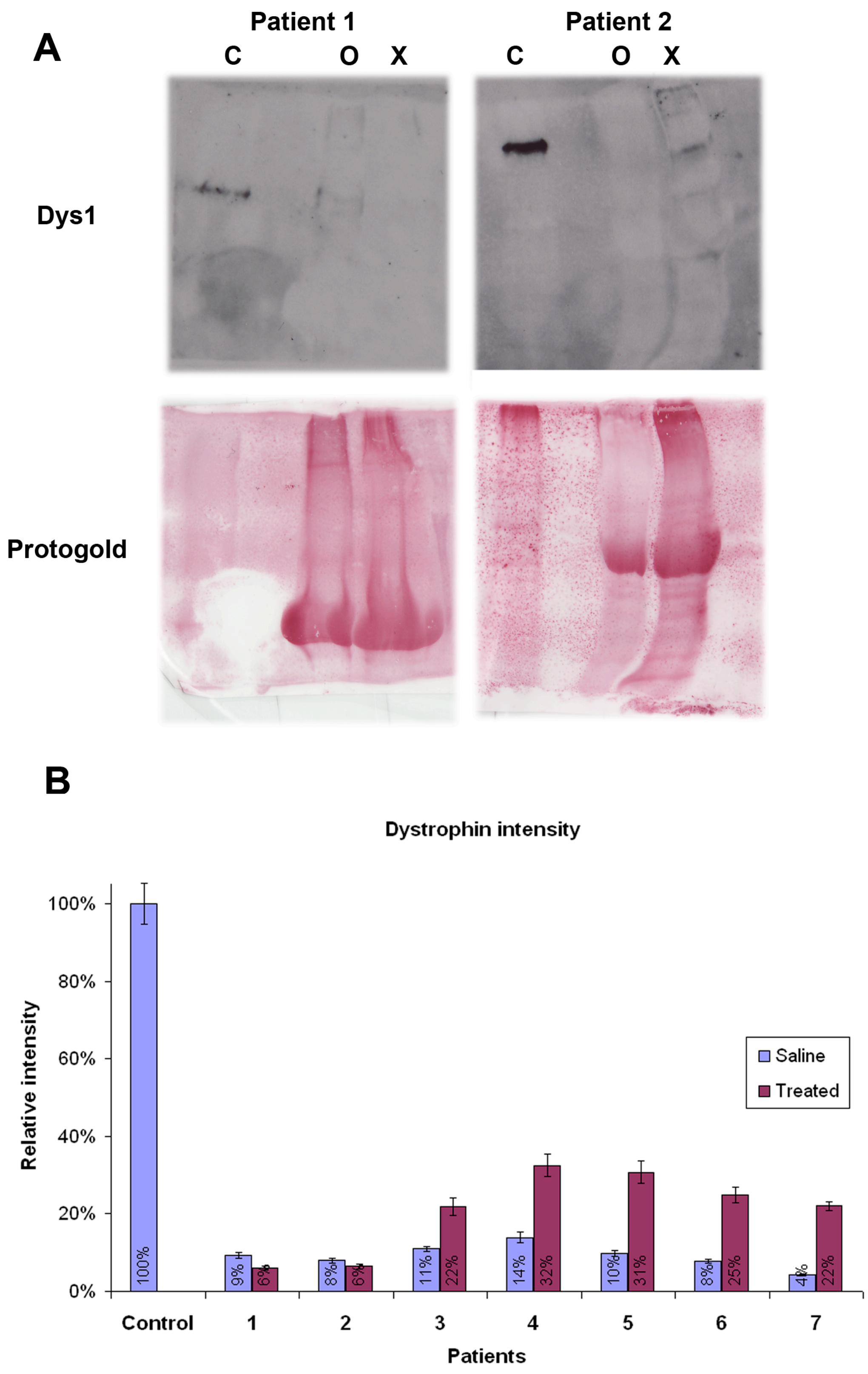
A
Patient 1
C
O
X
Patient 2
C
O
X
Dys1
Protogold
B
Dystrophin intensity
Relative intensity
100%
80%
60%
40%
20%
0%
Saline
Treated
100%
9%
6%
8%
6%
11%
22%
14%
32%
10%
31%
8%
25%
4%
22%
Control
1
2
3
4
5
6
7
Patients

***Supplementary Figure 4*: Protein expression between the treated and control EDBs in patients 1 and 2 (0·09mg AVI-4658)**

(A) Western blot analysis of homogenates of treated and untreated muscle (20x10µm sections) and control muscle (2x10µm sections) shows dystrophin expression in extracts from the control muscle (C) but not in the treated or untreated muscles from the patient. (B) Mean random intensity measurements: 4 fields of the immunostained transverse cryosection of each muscle were selected at random (out of focus) and were then photographed (in focus). Ten regions per image, each including an area of membrane and fibre cytoplasm, were selected by moving the cursor across the image and were analysed with MetaMorph. Analysis of average intensity showed an increase in dystrophin expression in patients 3–7 (p=0·002), but no increase was seen in patients 1 and 2 (who had the low dose [0·09 mg] AVI-4658). C=control muscle. O=untreated muscle. X=treated muscle.
